# Unraveling the Hidden Burden of Gastrointestinal and Nutritional Challenges in Children with Fabry Disease: A Systematic Review with Meta-Analysis

**DOI:** 10.3390/nu17071194

**Published:** 2025-03-29

**Authors:** Vanessa Nadia Dargenio, Maria Natale, Stefania Paola Castellaneta, Giovanni la Grasta, Leonardo Paulucci, Costantino Dargenio, Ruggiero Francavilla, Fernanda Cristofori

**Affiliations:** Interdisciplinary Department of Medicine, Pediatric Section, Children’s Hospital Giovanni XXIII, University of Bari Aldo Moro, 70126 Bari, Italy; vanessa.dargenio@unifg.it (V.N.D.); mariella.nat@live.it (M.N.); castellanetas@gmail.com (S.P.C.); g.lagrasta94@gmail.com (G.l.G.); leonardo.paulucci92@gmail.com (L.P.); costy.dargenio@gmail.com (C.D.); fernandacristofori@gmail.com (F.C.)

**Keywords:** Fabry disease, gastrointestinal symptoms, abdominal pain, diarrhea, children

## Abstract

Background/Objectives: Fabry Disease (FD) is a multisystem X-linked lysosomal storage disorder that often manifests with nonspecific gastrointestinal (GI) symptoms, such as abdominal pain, diarrhea, and constipation. These symptoms may appear early in childhood, severely impacting quality of life and delaying diagnosis, and may be linked to nutritional challenges. This systematic review aims to evaluate the prevalence, characteristics, clinical relevance, and nutritional aspects of GI manifestations in pediatric FD patients to aid in early recognition and improve outcomes. Methods: A systematic literature search with meta-analysis adhering to PRISMA and MOOSE guidelines was conducted across PubMed, Web of Science, and Google Scholar from inception to November 2024 using fixed inclusion and exclusion criteria. Data were extracted by two reviewers independently. Disagreements were resolved by consensus; a third reviewer was consulted, when necessary. Pooled analysis was performed by a random-effects model; heterogeneity was assessed using the I^2^ method. A quality assessment appraisal of the studies was carried out using the ROBINS-I tool. Results: The review encompassed 18 studies involving 736 pediatric patients. The evaluation of the pooled prevalence of GI symptoms in FD patients was 53% (95% CI 38–68%, I^2^ 90%), with abdominal pain being the most frequent (pooled prevalence of 46% (95% CI 33–60%, I^2^ 86%)). Symptoms often presented early, with a summarized standardized mean difference between the mean age of symptom onset and the mean age at FD diagnosis of 2.07 years (95% CI of 0.56–3.57, I^2^ 42%, *p* < 0.01). Nutritional issues, including reduced food intake and potential malabsorption, were reported in cases with severe GI symptoms, contributing to growth impairments. Conclusions: GI symptoms frequently constitute the earliest clinical manifestation of FD in children. Their nonspecific nature underscores the importance of heightened clinical suspicion for timely diagnosis. Early intervention, including enzyme replacement therapy and tailored nutritional strategies, can alleviate symptoms, improve quality of life, and prevent disease progression. Multidisciplinary approaches are essential to optimize patient outcomes and further research into the pathophysiology and management of GI symptoms in FD is warranted.

## 1. Introduction

Gastrointestinal (GI) symptoms of unknown origin represent a significant diagnostic challenge for gastroenterologists, pediatricians, surgeons, and general practitioners. The differential diagnosis is extensive, encompassing relatively common medical conditions including irritable bowel syndrome (IBS) and inflammatory bowel disease (IBD), as well as rare inherited metabolic illnesses such as Fabry disease (FD) [1].

FD is a multisystem and heterogeneous X-linked lysosomal storage disorder triggered by mutations in the alpha-galactosidase (GLA) gene, resulting in reduced or absent GLA enzymatic activity [2,3]. Consequently, neutral glycosphingolipids, predominantly globotriaosylceramide (GL3) and its derivatives, gradually accumulate in the lysosomes of different tissues, mainly in the vascular endothelium of the skin, kidney, heart, and nervous system, leading to progressive multiorgan dysfunction that compromises life expectancy [4,5].

Initially considered rare, with an estimated prevalence of 1 in 40,000 live births [6], recent evidence from newborn screening programs suggests that FD may affect as many as 1 in 3400–4000 newborns, particularly when including late-onset and milder GLA variants [6]. While both sexes can be affected, clinical manifestations are generally milder and later in onset in female carriers compared to affected males [7]. This is almost certainly in part due to the effects of X-chromosome inactivation, but it is also necessary to consider the potential genetic variability caused by the more than 1000 known mutations that have been reported in the literature, as well as the still-uncertain role that intronic mutations and single nucleotide polymorphisms play [8].

FD phenotypes vary widely, ranging from the classic presentation—characterized by angiokeratoma, acroparesthesias in childhood, and later-onset cardiovascular, neurological, and renal complications—to adult-onset variants with isolated cardiac or renal involvement [9].

Among the early manifestations of FD, GI symptoms are increasingly recognized as clinically significant. Studies indicate that most FD patients experience GI complaints, including abdominal pain and altered bowel habits [10]. While not typically life-threatening, these symptoms substantially impact patients’ quality of life and may serve as the earliest clinical indicators of FD. Due to their nonspecific nature and uncertain prevalence, timely diagnosis requires a high index of clinical suspicion.

Since the introduction of enzyme replacement therapy (ERT) in 2001, significant progress has been made in FD management. ERT has been shown to preserve organ function [11], decrease the severity of symptoms [12], and enhance quality of life [13]. Emerging research also suggests its potential role in alleviating GI symptoms, warranting further investigation into its impact on the digestive manifestations of FD. Awareness of FD as a potential underlying cause of nonspecific GI symptoms enables appropriate diagnostic investigations and facilitates the early initiation of disease-modifying treatments where required [10].

Nutritional therapy plays a vital role in the multidisciplinary management of FD, addressing GI and renal complications while targeting systemic inflammation and oxidative stress. When combined with ERT, tailored dietary interventions may enhance treatment outcomes, particularly in pediatric populations.

This study aims to systematically review the literature on GI symptoms in pediatric FD patients to evaluate their clinical significance as early manifestations of the disease and provide insights for improving early diagnosis and treatment, with a specific focus on addressing the nutritional requirements of children affected by FD.

## 2. Materials and Methods

### 2.1. Search Strategy

This systematic review with meta-analysis adheres to the 2020 Preferred Reporting Items for Systematic Reviews and Meta-Analyses (PRISMA) guidelines and Meta-analysis of Observational Studies in Epidemiology (MOOSE) guidelines [14,15]. A systematic search was conducted across the PubMed and Web of Science Core Collection (Clarivate Analytics) databases and Google Scholar, covering the period from database inception to November 2024. To ensure the rigor of selected studies, the search was limited to articles published in English in peer-reviewed journals.

A query structure based on Boolean combinations of the terms “Fabry disease”, “gastrointestinal”, “symptoms”, and “child”, with term variations, was used. For Google scholar, the search filter “only scientific articles” was also applied. The complete search strategies are described in detail in Supplementary Box S1.

Two authors (V.N.D. and M.N.) independently screened the titles, abstracts, and full texts of identified articles. Disagreements were resolved by consensus, and a third reviewer (R.F.) was consulted, when necessary.

### 2.2. Data Collection

We included articles fulfilling the following criteria: original articles and case series with more than three patients reporting GI manifestations in pediatric patients (until 18 years old) with FD of any gender and ethnicity. Additionally, articles with details regarding general symptoms (of which we chose the digestive ones) were included. These criteria were established to capture a comprehensive range of clinical presentations and enhance understanding of GI involvement in pediatric FD.

Studies were excluded if (1) they were review articles, as they do not provide original patient data; (2) they were articles published in languages other than English, to ensure accessibility and consistency in data interpretation; (3) they were articles with only an abstract available, due to insufficient methodological details. Finally, pediatric patients on ERT were excluded to specifically focus on the natural history and early presentation of GI symptoms in FD before treatment intervention.

### 2.3. Data Extraction and Management

A standardized data extraction sheet created in Microsoft Excel was used to collect relevant information. The extracted indicators were selected based on their relevance with respect to understanding the clinical significance of GI symptoms in pediatric FD. These included study characteristics, patient demographics, clinical presentations, diagnostic methods, and reported outcomes. Two authors (M.N. and V.N.D.) independently extracted data to ensure accuracy and reduce bias. The data extraction process was supervised and approved by two senior authors (F.C. and R.F.). In cases where critical data were missing, corresponding authors were contacted via email to request additional details. Any data that remained unavailable were recorded as N/A (not available).

### 2.4. Quality and Bias Assessment

The methodological quality of each study was assessed by two investigators (V.N.D. and F.C.) using the ROBINS-I tool for nonrandomized studies, version 2025 [16]. Any discrepancies in quality assessment were resolved by discussing and involving a third experienced arbitrator (R.F.).

### 2.5. Data Analyses

Dichotomous variables were expressed as a proportion with a 95% confidence interval (95% CI), while continuous variables were expressed as mean ± standard deviation (SD). To estimate the prevalence of GI symptoms, proportions were extracted from individual studies and compared across different study types. Where possible, a weighted average approach was used, giving greater weight to studies with larger sample sizes. Data from individual studies were pooled using the DerSimonian and Laird random-effects model [17]. Heterogeneity among studies was assessed using the I^2^ statistic, with cut-off points of <25%, 25–50%, 50–75%, and >75%, indicating little, low, moderate, and high heterogeneity [18].

Statistical analyses and graphics were conducted using a specific tool for meta-analysis [19]. Statistical significance was set at a threshold of α = 0.05.

Given the heterogeneity of the included studies in terms of design, sample size, and outcome measures, a narrative synthesis approach was used to integrate the findings. Study characteristics and results were also summarized descriptively, categorizing data based on symptom prevalence, age at onset, and gender-specific variations. The synthesis was structured to highlight trends and differences across study populations.

## 3. Results

**Figure 1:**
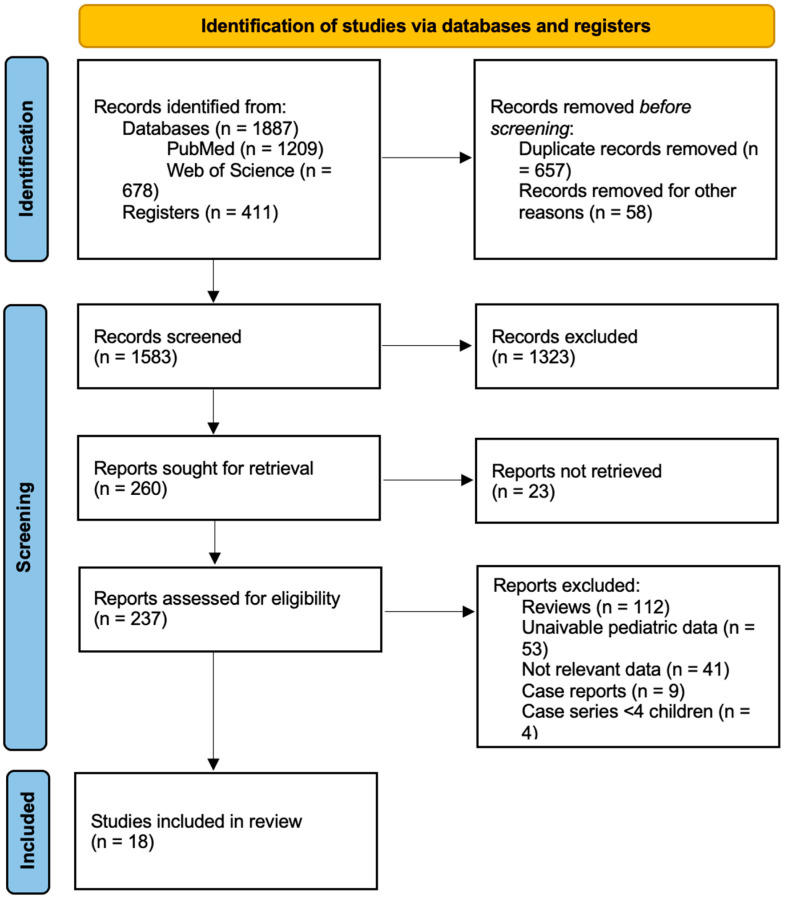
PRISMA flow chart for study retrieval and selection.

The flow diagram of the selection process is depicted in Figure 1.

After removing duplicates, 1583 articles were left for additional examination. Of these, 1323 were excluded based on their abstract and title, leaving 260 papers for full-text evaluation of eligibility. Fifty-three articles were excluded from the final database because, despite containing pediatric data, they did not distinguish between adult and pediatric populations. Nine case reports and four case studies with less than four patients were excluded from the analysis. Ultimately, 18 papers involving pediatric patients, published between 2001 and 2023, met the eligibility criteria and were included in our review. Of these, four were case studies including pediatric patients and, in the prospective studies, baseline characteristics were considered.

Each study’s design, outcomes, and relevant findings are reported in the Table 1.

Altogether, the 18 selected studies included a total of 736 FD patients from different geographical areas. Among these, five were cross-sectional [4,20,21,29,31], three were retrospective [11,24,30], six were prospective [9,22,23,25,27,28] and four were case series with more than 4 patients [26,32,33,34]. The number of patients ranged from 4 to 352.

The summarized mean age at onset of GI symptoms in FD patients was 8.5 years (95% CI 5.4–11.59, I^2^ 97%), while the summarized mean age at diagnosis was 10.7 years (95% CI 9.8–11.69, I^2^ 97%). The summarized standardized mean difference between the mean age at symptom onset and the mean age at FD diagnosis was 2.07 years (95% CI 0.56–3.57, I^2^ 42%, *p* < 0.01).

**Figure 2:**
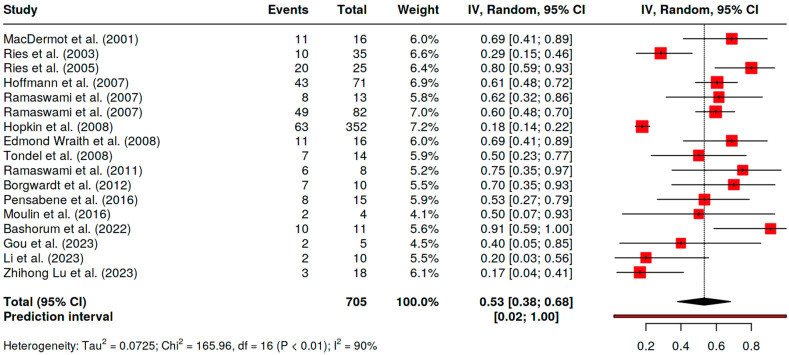
Pooled prevalence of GI symptoms in FD children [4,9,11,20,21,22,23,24,25,26,27,29,30,31,32,33,34].

The evaluation of the pooled prevalence of GI symptoms in FD patients was 53% (95% CI 38–68%, I^2^ 90%) (Figure 2).

Cohort studies have highlighted that GI involvement is prevalent in up to 70–80% of children with FD [4,20,25,27]. The lowest prevalence reported was 18% [24], with males typically presenting symptoms earlier and more severely than females [20,24]. GI symptoms commonly manifest in early childhood, with a median onset age of five years for males and 9.5 years for females according to the largest registry reviewed [24]. Symptoms have been observed in children as young as 1–4 years, with abdominal discomfort being the most reported complaint [9,23,24,35].

The most frequently reported GI symptom was abdominal discomfort, with a pooled prevalence of 46% (95% CI 33–60%, I^2^ 86%) (Supplementary Figure S1), ranging from 26.7% to nearly 70% [21,24,30]. It was characterized as a burning pain or colic that affects the entire abdomen or just the mid and lower part, with tenderness at palpation, and that may be brought on by eating or worsened when altering eating habits. Concerning the relation with gender, there were no differences between males and females in the large cohorts investigated by Hoffman et al. and Ramaswami et al. [9,22]

Diarrhea was the second most reported symptom, with a pooled prevalence of 31% (95% CI 22–41%, I^2^ 73%) (Supplementary Figure S2), affecting between 19.3% and 48% of children [21,24], with a higher prevalence in males (25.9–33%) than females (16.7–27.6%) [9,22]. Episodes could follow meals, especially those with high-fat foods, and were characterized by urgency to defecate, frequent loose stools, and cramping.

In contrast, constipation was reported in 16% of patients (95% CI 8–25%, I^2^ 55%) (Supplementary Figure S3). Gender-specific prevalence varied across studies: one study reported a higher prevalence in females than males (16.7% vs. 8.6%) [22], while another study found the opposite (6.9% in females vs. 13.9% in males) [9].

Additional symptoms include bloating, epigastric discomfort, and early satiety after meals, resembling irritable bowel syndrome (IBS). Laboratory tests and endoscopy in early FD stages typically show normal results, despite persistent symptoms. Some patients experience alternating patterns of diarrhea and constipation, with abdominal pain mimicking IBS [36].

Less commonly reported symptoms include nausea [18%, 95% CI 13–24%, I^2^ 0%; (Supplementary Figure S4)] and vomiting (12%, 95% CI 8–17%, I^2^ 0%) (Supplementary Figure S5), primarily among males [9,22].

Although not included in the systematic review analysis, we preferred to report data from case reports and case series with fewer than four patients in Table 2 to provide a more comprehensive framework for the reader.

Regarding rare cases, an overlap with achalasia and phenylketonuria in FD patients was reported in the literature [35,38].

Case studies have highlighted severe outcomes such as jejunal perforation and chronic pseudo-obstruction syndrome [41,43], with clinical improvement noted following ERT. Other reports describe delayed gastric emptying [37], postprandial fullness [44], and overlapping autoimmune conditions such as celiac disease [39]. These findings emphasize the complexity and variability of GI symptoms in FD patients.

### Quality Assessment and Risk of Bias

**Figure 3:**
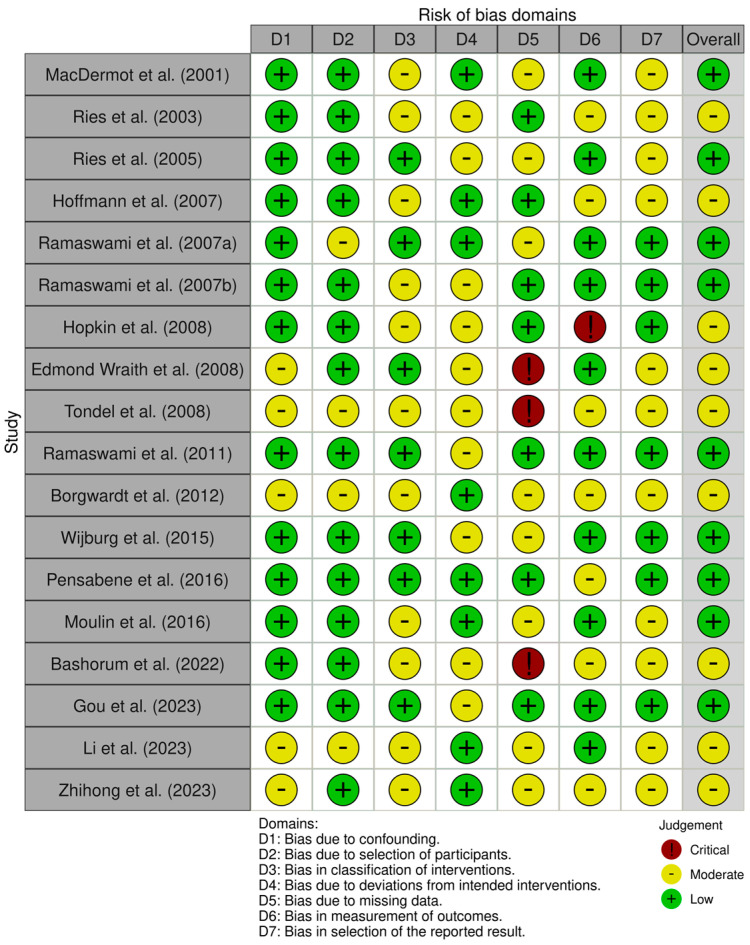
ROBINS-I assessment of the risk of bias in the included studies [4,9,11,20,21,22,23,24,25,26,27,28,29,30,31,32,33,34].

The methodological quality of studies ranged from low to critical. The overall risk of bias was low in nine (50%) [4,9,11,21,23,28,29,30,32] and moderate in nine (50%) [20,22,24,25,26,27,31,33,34] (Supplementary Figure S6). In detail, the risk of bias was low in 8 (44.5%) [4,22,23,27,29,30,33,34] and moderate in 10 (55.5%) [9,11,20,21,24,25,26,28,31,32] for deviation from intended intervention; low in 7 (38.9%) [9,11,20,22,24,29,32], moderate in 8 (44.4%) [4,21,23,27,28,30,33,34], and critical in 3 (16.7%) [25,26,31] for missing outcomes; low in 10 (55.5%) [4,9,11,21,23,25,28,30,32,33], moderate in 7 (38.9%) [20,22,26,27,29,31,34], and critical in 1 (0.6%) [24] for measurements of outcomes; and low in 7 (38.9%) [9,11,23,24,28,29,32] and moderate in 11 (61.1%) [4,20,21,22,25,26,27,30,31,33,34] for the selection of reported results (Figure 3).

## 4. Discussion

FD is a chronic, progressive, multisystemic genetic disorder characterized by a broad spectrum of manifestations. This systematic review highlights that GI symptoms are among the first warning indicators of FD in young patients, affecting up to two-thirds of children and having the potential to occur in the earliest years of life. The most frequently reported GI symptom is abdominal pain, followed by diarrhea, constipation, nausea, and vomiting, which are unspecified functional bowel disorders. These symptoms vary in incidence by age and gender, with males generally experiencing earlier and more severe GI involvement compared to females. Clinical presentations can range from a single severe symptom to a combination of multiple symptoms impacting the health and daily functioning of affected children.

The GI symptoms of FD are caused by a complex and multifaceted pathophysiology. It is believed that accumulation begins during pregnancy since GL3 is present in the placenta and fetal tissue, including renal and cardiac cells [43,49]. Three main processes appear to be involved: vasculopathy that affects GI circulation, tissue inflammation linked to GL3 deposits, and dysfunction of the autonomic nervous system, which controls gut motility [50,51].

An increase in pain intensity with increasing metabolic demand supports the theory that abdominal discomfort is neuroischemic in nature, resulting from insufficient blood supply to the GI tract. Furthermore, abdominal pain, gastroparesis, and abnormal intestinal mobility are probably caused by small-fiber neuropathy, resembling peripheral neuropathy that causes acroparesthesias [43,50]. Additionally, GL3 deposits within the villi may be a contributing factor in the diarrhea, causing inflammation, a reduction in villi activity, and eventual malabsorption [50,52]. Increased sphingolipid accumulation in the ganglion cells of the autonomic nervous system that innervate the esophagus and hindgut have been found in autopsies, supporting the theory that upper GI symptoms are caused by substrate accumulation leading to neuronal dysfunction [50]. Diverticula in the duodenum, jejunum, and colon have also been demonstrated to result from high intraluminal pressure caused by dysmotility, with serious and perhaps lethal outcomes [43,50], with increased risk of infection and perforation. Achalasia probably also has the same etiopathogenesis, due to the GL3 accumulation in the esophageal myenteric plexus, leading to disordered esophageal motility.

Early onset of GI symptoms, even in children aged 1–4 years, was previously emphasized in a 2015 systematic review by Laney et al. [53]. Nonetheless, symptom onset appears highly variable, even within families, reflecting considerable inter- and intra-family phenotypic variability. This variability likely results from factors beyond GLA gene mutations, including environmental and other genetic influences [54]. A recent study conducted by Di Martino et al. identified an association between GI symptoms in FD patients and several single nucleotide polymorphisms in ADME-related genes involved in bile acid detoxification, export, and absorption in the liver [54]. The authors suggested that genetic variability in these genes might be connected to vulnerability to GI symptoms, notably diarrhea, through altered enterohepatic bile acid circulation [54].

GI symptoms may occur immediately after the development of acroparesthesias. Nevertheless, according to several FD registries, GI symptoms can be the initial symptom in nearly a fourth of the boys with FD, present at the age of 5 years, and in about a tenth of the girls, starting at the age of 9 years [20,24]. Additionally, Hoffmann et al. observed that complaints related to abdominal pain and diarrhea decreased with age at diagnosis, from 49.3% and 25.4% in pediatric age to 38% and 19.2% in adulthood, respectively [22]. In comparison, the prevalence of abdominal pain was 13.5% and diarrhea was 19.2% among healthy children [55].

GI manifestations can also impair growth and nutritional status in affected children, with males more likely to be underweight and shorter for their age compared to females [56]. Stress or meal-related symptom exacerbations may lead to reduced food intake and weight loss, though these effects are generally observed in patients with severe symptoms. Hoffman et al. reported no significant differences in body mass index (BMI) between FD patients with and without GI symptoms, highlighting variability in nutritional impact [13].

Patients with FD can go years without receiving a diagnosis because of the variable clinical presentation. Diagnoses may be delayed by three to nearly twenty years between the onset of symptoms and confirmed diagnosis [4].

We observe that patients who present with a long-term history of unexplained GI symptoms, such as postprandial abdominal pain, non-inflammatory diarrhea with frequent urgency, early satiety or gastroparesis, or chronic intestinal pseudo-obstruction, should be evaluated for FD as the possible cause of their GI issues. Other symptoms associated with FD, such as neuropathic pain (especially burning pain in the hands and feet), impaired sensations of warmth and cold, and subtle autonomic nervous system dysfunction (such as heat intolerance or abnormal sweating), should also raise suspicion. Ultimately, medical professionals need to determine if the patient has any dermatological, ophthalmic, renal, or cardiac problems, a long family history of these symptoms, or FD itself [57,58,59]. Tests carried out as part of the assessment of nonspecific GI symptoms might help distinguish FD from other common GI conditions. However, for patients with unexplained GI symptoms and unclear diagnoses, if there is a clinical suspicion of FD, confirmatory diagnosis is required, based on enzyme and/or genotype testing. For a timely diagnosis, clinicians should be aware of any GI symptoms that may be connected to this illness. In fact, MacDermot et al. conducted a survey of patients with GI symptoms and undiagnosed FD, showing that these patients had undergone a variety of investigations before receiving their diagnosis, including barium meals, gastroscopies, and colonoscopies, which frequently revealed no abnormalities [4]. However, GI tract biopsies may support diagnosis in uncertain cases, revealing positive anti-GL3 immunostaining results [43,51].

In FD, differential diagnosis is crucial, particularly for patients who report nonspecific GI symptoms. It is necessary to conduct investigations to rule out more common conditions that may be mistaken for FD, such as diverticular disease, appendicitis, celiac disease, gastritis, irritable bowel syndrome, appendicitis, colon cancer, and gastro-esophageal reflux. Other uncommon illnesses may also mimic FD, such as Whipple’s illness, transthyretin-related familial amyloid polyneuropathy, or mitochondrial disorders [10]. To date, there are no established guidelines that offer recommendations for GI tract examinations in children with FD. Furthermore, there are not any validated tools available that are designed expressly to evaluate GI symptoms in FD. Shields et al. and Hilz et al. have proposed questionnaires to evaluate these patients, but while showing potential benefit in clinical trials, they have not yet been validated and should be implemented in clinical practice, considering that they are inexpensive, simple to use, and maybe even life-saving [10,60].

Regular monitoring of GI symptoms, including weight changes, bowel habits, and dietary history, should be integrated into FD management at diagnosis and repeated annually. Radiologic or endoscopic analysis may be helpful to exclude non Fabry-related causes of severe abdominal pain [61].

Patients with persistent GI symptoms experience substantial impairment in physical, emotional, and social well-being [24]. Chronic abdominal pain and diarrhea can lead to malnutrition, weight loss, and growth impairment, particularly in males. Moreover, GI distress can disrupt school attendance and limit social participation, further affecting psychological well-being [25].

Finally, treatment should begin promptly to alleviate symptoms and prevent long-term complications. The most frequently used treatments are ERT (with either alfa or beta galactosidase) and chaperone therapy. It has been demonstrated that prompt ERT start, beginning as soon as feasible after diagnosis, improves long-term renal and cardiac outcomes in addition to other clinical outcomes and slows the course of the illness in FD children [62,63]. Specifically, research suggests that starting ERT early in children can repair kidney damage that is irreversible by the time clinical indications of renal impairment appear [1,2,25,43,51,52,53]. This therapy may even improve, up to regression, the GI symptoms associated with FD, with fewer school absences due to the illness and improvement in the quality of life of patients [9,22,43,64]. Additional symptom-specific treatments include loperamide for diarrhea, though long-term use should be monitored for constipation, prokinetics such as metoclopramide for gastroparesis, and proton pump inhibitors for gastroesophageal reflux. While these medications offer symptomatic relief, their effectiveness varies, and their adverse effects may limit prolonged use.

Non-pharmacological interventions, such as dietary modifications, should be explored, though patient adherence remains a challenge [37,65,66].

Emerging evidence suggests that implementing a low-FODMAP (fermentable oligosaccharides, disaccharides, monosaccharides, and polyols) diet may alleviate these GI disturbances [56,67]. This dietary approach reduces the intake of fermentable carbohydrates, which exacerbates symptoms by increasing colonic gas production and intestinal water content. While studies have demonstrated the efficacy of this diet in managing IBS, its application in FD warrants further clinical investigation to confirm its benefits and establish specific guidelines for this patient cohort [68].

Renal involvement in FD, often culminating in chronic kidney disease (CKD), necessitates precise dietary management to slow disease progression [69]. Protein intake is critical; excessive protein consumption exacerbates albuminuria and accelerates nephron damage [70]. Therefore, a low-protein diet, typically ranging from 0.6 to 0.8 g per kg of body weight per day, is recommended to preserve renal function [71]. Additionally, sodium restriction, with an intake of less than 3 g per day, is advised to manage hypertension and reduce albuminuria [72]. This strategy mitigates renal complications and addresses the heightened cardiovascular risks associated with FD nephropathy. Hydration status is crucial in supporting renal function and minimizing dehydration-related complications, especially in children with increased renal involvement [73]. Fluid intake recommendations should be tailored to the individual patient’s clinical status, particularly in those with compromised renal function.

Dietary interventions incorporating anti-inflammatory and antioxidant-rich foods are emphasized to combat systemic inflammation and oxidative stress, which are central to the pathogenesis of FD [74,75]. The Mediterranean diet, characterized by high levels of omega-3 fatty acids, polyphenols, and dietary fiber, has demonstrated efficacy in reducing inflammatory markers and improving cardiovascular outcomes in patients with CKD [76]. This dietary pattern may also benefit pediatric FD patients by mitigating the inflammatory processes triggered by GL3 accumulation, particularly through its influence on oxidative stress and endothelial dysfunction [71].

Nutritional monitoring in children with FD is imperative to ensure proper growth and development while preventing malnutrition. Although GI involvement in FD rarely results in malabsorption, regular assessments of nutritional biomarkers such as serum protein, albumin, calcium, and vitamins (e.g., B12 and folate) are recommended [77]. In cases where deficiencies are identified, targeted supplementation should be implemented [71]. Probiotics and prebiotics remain potential adjuncts to address dysbiosis and promote gut health; however, their clinical utility in FD remains to be conclusively established [78]. Further studies are necessary to determine their role in modulating gut microbiota and their overall impact on GI symptom management in FD patients.

In conclusion, nutritional therapy constitutes an integral component of the multidisciplinary management of FD in children. By addressing GI symptoms, renal dysfunction, and systemic inflammation, dietary strategies enhance the effectiveness of enzyme replacement therapy and improve overall patient well-being. Further research is warranted to refine these interventions and explore their long-term impact on disease progression and quality of life. The application of evidence-based nutritional principles tailored to the unique needs of pediatric FD patients underscores the importance of an individualized approach to care.

Beyond dietary modifications, additional non-pharmacological approaches should be considered. Cognitive behavioral therapy has been shown to help patients with chronic GI conditions, particularly those with functional bowel disorders [79]. Given the overlap between FD-related GI symptoms and irritable bowel syndrome, behavioral therapy may improve coping strategies and reduce symptom severity. Used in IBS management, hypnotherapy may be beneficial in FD patients experiencing chronic pain and dysmotility. Regular exercise has been found to support gut motility and may be particularly beneficial in patients with FD-related dysmotility. Although these interventions require further study in FD-specific populations, their implementation could enhance symptom control and improve quality of life [79].

Although there is still a long way to go before gene treatments are clinically implemented for FD, they have shown promise as novel therapy mechanisms [80].

For now, advances in symptom management and early diagnosis offer hope for improved outcomes. Expanding clinician awareness of FD as a potential etiology of nonspecific GI symptoms will be key to timely diagnosis and intervention, ultimately enhancing the quality of life for affected patients and their families.

## 5. Strengths and Limitations

This review has several strengths. It is the first systematic review with meta-analysis to comprehensively assess the prevalence, characteristics, and impact of GI manifestations in pediatric patients with FD. By focusing exclusively on pediatric populations, it highlights the early onset of symptoms and the need for heightened clinical suspicion in this age group. The adherence to PRISMA and MOOSE guidelines and use of standardized tools for data extraction and quality assessment enhance the transparency and reproducibility of the review.

The evidence included in this systematic review is subject to several limitations. First, the majority of studies were observational, such as cross-sectional or retrospective designs, which inherently limit the ability to establish causality between FD and its GI manifestations. For instance, some studies lacked a detailed stratification of GI symptoms by age or gender. Additionally, many studies relied on small sample sizes, particularly case series, which may limit the generalizability of their findings. Furthermore, another critical limitation is the variability in how GI symptoms were assessed and reported, with some studies relying on patient self-reports or non-standardized tools, which may introduce reporting bias. Lastly, the exclusion of studies involving adult populations restricts the ability to compare the progression of GI symptoms across different age groups.

In conclusion, while this systematic review with meta-analysis provides valuable insights into the GI manifestations of FD in pediatric populations, the limitations of the evidence and review process underscore the need for more robust, standardized, and longitudinal studies to improve our understanding of this condition.

## 6. Conclusions

GI symptoms are highly prevalent in pediatric patients with FD and often represent the earliest clinical manifestation of the condition. Symptoms such as abdominal pain, diarrhea, constipation, nausea, and vomiting severely impact the quality of life and daily activities of affected children. Due to their nonspecific nature and overlap with other GI disorders, these symptoms frequently contribute to diagnostic delays, postponing the initiation of effective treatment.

Clinicians managing pediatric patients with persistent and unexplained upper or lower GI symptoms should consider FD as a potential diagnosis, particularly in cases that do not respond to standard therapies for FGIDs. A detailed clinical evaluation, including family history and physical examination, is essential for identifying potential red flags of FD, such as neuropathic pain. Advances in diagnostic methodologies, including genetic testing and validated symptom assessment tools, now offer effective strategies for early detection. In cases of diagnostic uncertainty, temporary symptom management strategies, such as dietary modifications and symptomatic relief with antispasmodics or anti-diarrheal agents, can help improve patient comfort while awaiting confirmatory testing.

Timely diagnosis and the initiation of targeted treatments, such as ERT or pharmacological chaperones, are critical to halting disease progression, preventing irreversible organ damage, and improving long-term outcomes. Early intervention not only enhances the quality of life for affected children but also alleviates the emotional and psychosocial burden on their families.

A multidisciplinary approach—bringing together pediatric gastroenterologists, geneticists, dietitians, and other specialists—is crucial to improving the diagnostic process and optimizing care. Continued research into the GI manifestations of FD will further refine diagnostic criteria and inform evidence-based treatment protocols. Increased awareness among healthcare professionals about FD as a potential cause of nonspecific GI symptoms in children will facilitate earlier diagnosis and intervention, ultimately leading to better clinical outcomes for both patients and their families.

Finally, nutritional therapy is a critical adjunct to ERT in the management of FD disease, particularly in children. Targeted dietary interventions should be tailored to the stage of disease progression and specific patient needs. A low-FODMAP diet is recommended in patients with significant bloating, abdominal pain, and diarrhea, as it reduces colonic fermentation and gas production. In contrast, a low-protein diet (0.6–0.8 g/kg/day) is advised in patients with renal involvement to minimize nephron damage and reduce albuminuria. Anti-inflammatory and antioxidant-rich dietary patterns, such as the Mediterranean diet, may benefit patients across all disease stages by mitigating systemic inflammation and oxidative stress. Regular nutritional monitoring and tailored supplementation ensure proper growth and development while addressing disease-specific complications. Further research is needed to establish evidence-based dietary guidelines specific to FD and to assess the long-term impact of these interventions on disease progression and patient outcomes.

Future studies should focus on the long-term effects of nutritional therapy, its role in modifying disease progression, and its interaction with pharmacological treatments. Investigating the gut microbiota’s involvement in FD-related GI symptoms could provide novel therapeutic targets. Additionally, standardized symptom assessment tools and dietary intervention protocols should be developed to enhance clinical applicability. Expanding research efforts in these areas will refine treatment strategies, leading to improved symptom control and long-term prognosis for pediatric FD patients.

## Figures and Tables

**Table 1:** Main characteristics of the studies included in the systematic review.

Reference	Study Design	Patients (n)	Age in y (Mean ± SD, Median [Range])	Sex (M/F)	Main GI Symptoms	Age at Onset of GI Symptoms (Mean ± SD, Median [Range])	Number of Patients with GI Symptoms (%)	Overall Prevalence of GI Symptoms (%)	Age at Diagnosis in y (Mean ± SD, Median [Range])
MacDermot et al. (2001) [4]	Cross-sectional	16 children out of a cohort of 70	/	16 M	Abdominal pain, nausea, vomiting, post-prandial fulness	12	N/A	69%	/
Ries et al. (2003) [20]	Cross-sectional	35	12.6 [1–21]	15 M 20 F	Diarrhea, nausea, vomiting	N/A	17% (6 M/35)	28.6%	/
					Abdominal pain, constipation	N/A	11.5% (4 F/35)		
Ries et al. (2005) [21]	Cross-sectional	25	12.3 ± 3.5, 12 [5.6–18]	25 M	Abdominal pain	5 for M	72% (18/25)	80% (20/25)	/
					Diarrhea	9.5 for F	48% (12/25)		
Hoffmann et al. (2007) [22]	Prospective (FOS database)	71 children out of a cohort of 342	10.9 ± 5.0	57 M 14 F	Abdominal pain	14	49.3% (no difference between M and F)	60.8% (43/71)	/
					Diarrhea	15.5	25.4% (25.9% M; 16.7% F)		
					Constipation	17.5	13.3% (8.6% M; 16.7% F)		
					Nausea	12.7	15.5%		
					Vomiting	12.5	13.4%		
Ramaswami et al. (2007) [23]	Prospective	13	10.4 for M; 14.5 for F [2–18]	9 M 4 F	Abdominal pain	N/A	53.8% [4/9 M (44%); 3/4 F (75%)]	61.5% (8/13)	/
					Diarrhea		30.7% [3/9 M (33.3%); 1/4 F (25%)]		
					Constipation		30.7% [3/9 M (33.3%); 1/4 F (25%)]		
					Colic		23% [3/9 M (11%)]		
Ramaswami et al. (2007) [9]	Prospective (FOS database)	82	12.9 [0.7–17.9]	40 M 42 F	Abdominal pain	6.5 [1.0–15.4] for M 9.9 [1.7–17.8] for F	47.7% [16/36 M (44.4%); 15/29 F (51.7%)]	60%	/
					Constipation	9.6 [1.0–14.5] for M 6.6 [6.4–6.7] for F	10.7% [5/36 M (13.9%); 2/29 F (6.9%)]		
					Diarrhea	4.7 [1.0–13.1] for M 10.2 [1.0–15.4] for F	30.8% [12/36 M (33.3%); 8/29 F (27.6%)]		
					Nausea	7.2 [1.0–12.7] for M 14.8 [6.7–15.9] for F	23% [9/36 M (25%); 6/29 F (20.7%)]		
					Vomiting	6.5 [3.0–11.6] for M 15.6 [14.3–16.1] for F	10.8% [3/36 M (8.3%); 4/29 F (13.8%)]		
Hopkin et al. (2008) [24]	Retrospective (Fabry Registry)	352	12 [0–17]	194 M 158 F	Abdominal pain	5 y for M	26.7%	17.9% [45/194 M (23%); 18/158 F (11.3%)]	8.6 ± 7.5, 9 [<1−17]
					Diarrhea	9.5 y for F	19.3%		
Edmond Wraith et al. (2008) [25]	Prospective	16	12.1 ± 2.5, 11.7 [8.5–16]	14 M 2 F	Nausea, vomiting, post-prandial pain	N/A	N/A	68.8% (11/16)	12.1
Tondel et al. (2008) [26]	Case series	14	12	14 M 7 F	GI involvement	/	/	50% (7/14)	/
Ramaswami et al. (2011) [11]	Retrospective	8	5.0 ± 1.6 [0.4–6]	7 M 1 F	Abdominal pain	0.4–6	75% (6/8)	75% (6/8)	/
					diarrhea		37.5% (3/8)		
					constipation		37.5% (3/8)		
					vomiting		12.5% (1/8)		
Borgwardt et al. (2012) [27]	Prospective	10	[9–16]	6 M 4 F	Abdominal pain	N/A	/	70% (7/10)	7.3
Wijburg et al. (2015) [28]	Prospective	31	/	31 M	Abdominal pain	/	65% (20/31)	/	10 (0–17)
					diarrhea		32% (11/31)		
					nausea		29% (9/31)		
					vomiting		16% (5/31)		
Pensabene et al. (2016) [29]	Cross-sectional	15	11.8 [4–18]	5 M 10 F	diarrhea	10	53.3% (8/15)	53.3% (8/15)	/
					constipation		33.3% (5/15)		
					vomiting		13.3% (2/15)		
Moulin et al. (2016) [30]	Retrospective	4	10.5	3 M	Abdominal pain	10	50% (2/4)	50% (2/4)	10.5
Bashorum et al. (2022) [31]	Cross-sectional	11	/	/	Abdominal pain	/	90.9% (10/11)	90.9% (10/11)	/
					Diarrhea		81.8% (9/11)		
Gou et al. (2023) [32]	Case series	5	7 [1.1–13.1]	4 M 1 F	Abdominal pain	3.8	/	40%	4.5
Li et al. (2023) [33]	Case series	10	/	7 M 3 F	Abdominal pain, diarrhea	9	20% and 10%, respectively	20%	12
Zhihong et al. (2023) [34]	Case series	18	11.6 ± 2.7 [6–17]	14 M 4 F	Abdominal pain, diarrhea	/	/	16.6%	12 ± 2.7

F, female; FGID, functional abdominal pain; FOS, Fabry Outcome Survey; GI, gastrointestinal; IBS, irritable bowel syndrome; M, male; N/A, not available; n, number; SD, standard deviation; y, years.

**Table 2:** Main characteristics of Fabry Disease case reports and case series with fewer than four patients.

Reference	Study Design	Patients (n)	Age in y (Mean ± SD, Median [Range])	Sex (M/F)	Main GI Symptoms	Age at Onset of GI Symptoms (Mean ± SD, Median [Range])	Age at Diagnosis in y (Mean ± SD, Median [Range])
Argoff et al. (1998) [37]	Case series	1	3 y	M	Early satiety, post-prandial bloating, pain, vomiting (delayed gastric emptying)	3 y	N/A
Field et al. (2001) [38]	Case report	1	/	M	Dysphagia due to achalasia	15 y	15 y
Tumer et al. (2004) [39]	Case report	1	/	F	Celiac disease	11 y	11 y
Banikazemi et al. (2005) [40]	Case report	1	17 y	M	Abdominal pain, diarrhea, nausea, vomiting	15	/
Concolino et al. (2010) [35]	Case report	1	/	/	Phenylketonuria, abdominal pain, nonspecific episodes of gastroenteritis	21 months	3 y
Buda et al. (2011) [41]	Case report	1	/	M	Chronic pseudo-obstruction syndrome	9 y	15 y
Ellaway et al. (2014) [42]	Case series	2	/	2 F	Abdominal pain and nausea	12 y and 5 y	/
					Abdominal pain and constipation		
Politei et al. (2015) [43]	Case series	2	/	2 M	Abdominal pain, diarrhea and vomiting	6 y and 13 y	28 y and 17 y
Tuttolomondo et al. (2015) [44]	Case series	1	/	M	Abdominal pain, post prandial bloating and pain, early satiety, diarrhea and vomiting	9 y	9 y
Skrunes et al. (2017) [45]	Case series	2	/	1 M	Abdominal pain, diarrhea	5 y and 9 y	5 y and 9 y
				1 F	Abdominal pain		
Mhanni et al. (2020) [46]	Case series	2	/	2 M	Abdominal pain, nausea, diarrhea, constipation	N/A	10 y and 9 y
					Abdominal pain		
Onay et al. (2020) [47]	Case series	1	/	F	Abdominal pain, unable to gain weight	12 y	12 y
Paim-Marques et al. (2021) [48]	Case series	1	/	M	Abdominal pain, diarrhea	5 y	5 y

F, female; GI, gastrointestinal; M, male; N/A, not available; n, number; SD, standard deviation; y, years.

## Data Availability

All the data are available in the review.

## Supplementary Materials

The following supporting information can be downloaded at https://www.mdpi.com/article/10.3390/nu17071194/s1: Box S1: Search strategy; Supplementary Figure S1: Pooled prevalence of abdominal pain in FD children; Supplementary Figure S2: Pooled prevalence of diarrhea in FD children; Supplementary Figure S3: Pooled prevalence of constipation in FD children; Supplementary Figure S4: Pooled prevalence of nausea in FD children; Supplementary Figure S5: Pooled prevalence of vomiting in FD children; Supplementary Figure S6: Overall risk of bias of the included studies.
