# Supplementary material for: Unraveling the Hidden Burden of Gastrointestinal and Nutritional Challenges in Children with Fabry Disease: A Systematic Review with Meta-Analysis
Source: Nutrients. 2025 Mar 29;17(7):1194. doi: 10.3390/nu17071194 (PMC11990627; doi:10.3390/nu17071194)
Supplement: Supplementary file 1 [file nutrients-17-01194-s001.zip › supplementary/nutrients-3465364-supplementary.pdf]

Supplementary box 1. Search strategy

("Fabry Disease"[MeSH Terms] OR "Fabry Disease"[All Fields]) AND (("Child"[MeSH Terms] OR "Children"[All Fields] OR "Pediatrics"[All Fields]) OR ("Gastrointestinal Symptoms"[All Fields] OR "Gastrointestinal Manifestations"[All Fields]))
