# Supplementary figures and images for: Unraveling the Hidden Burden of Gastrointestinal and Nutritional Challenges in Children with Fabry Disease: A Systematic Review with Meta-Analysis
Source: Nutrients. 2025 Mar 29;17(7):1194. doi: 10.3390/nu17071194 (PMC11990627; doi:10.3390/nu17071194)

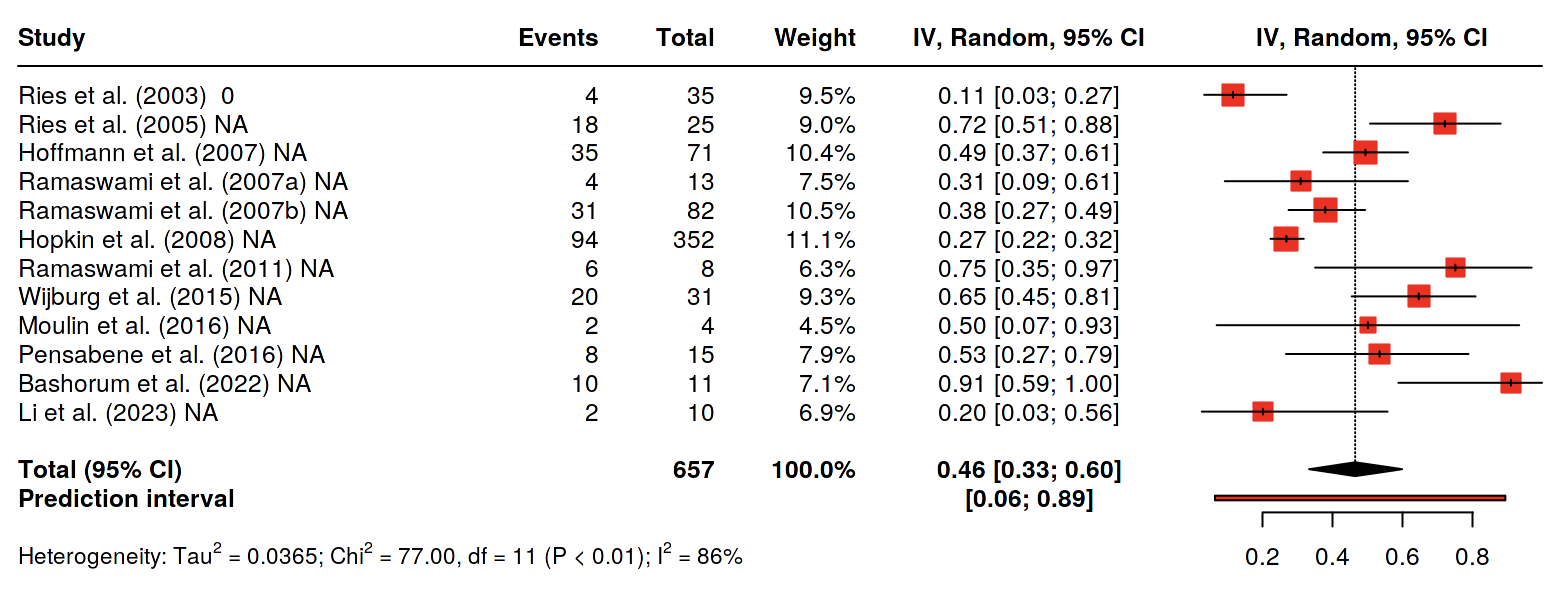

Supplement: Supplementary file 1 [file nutrients-17-01194-s001.zip › supplementary/supplementary Figure S1.png]

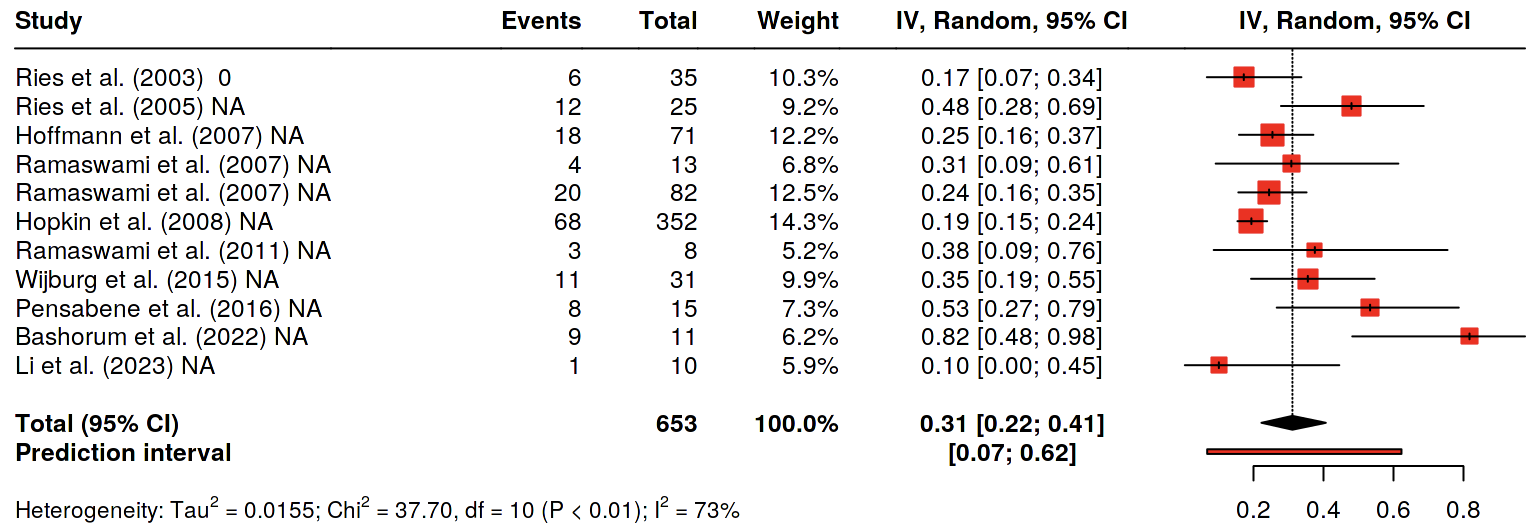

Supplement: Supplementary file 1 [file nutrients-17-01194-s001.zip › supplementary/supplementary Figure S2.png]

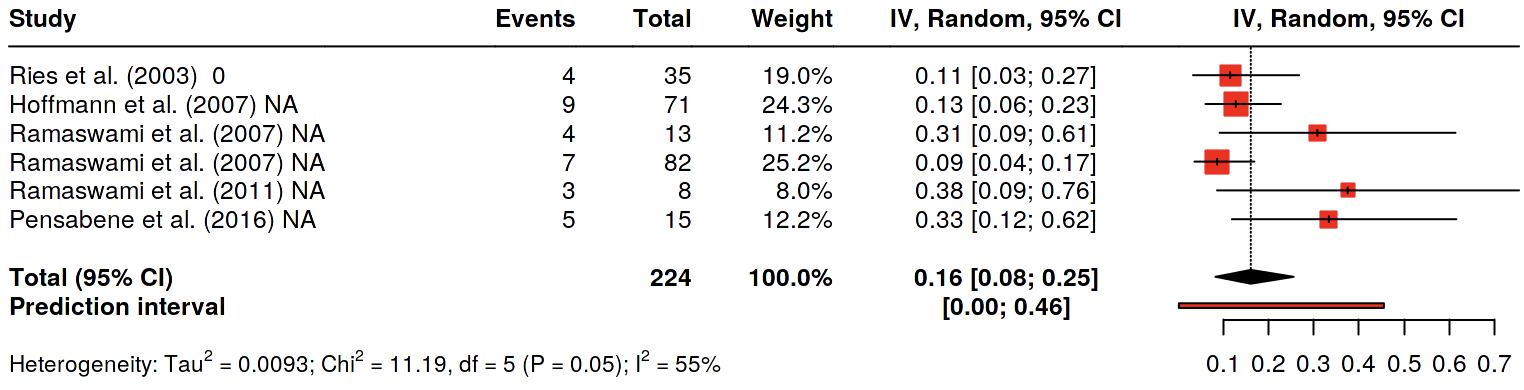

Supplement: Supplementary file 1 [file nutrients-17-01194-s001.zip › supplementary/supplementary figure S3.png]

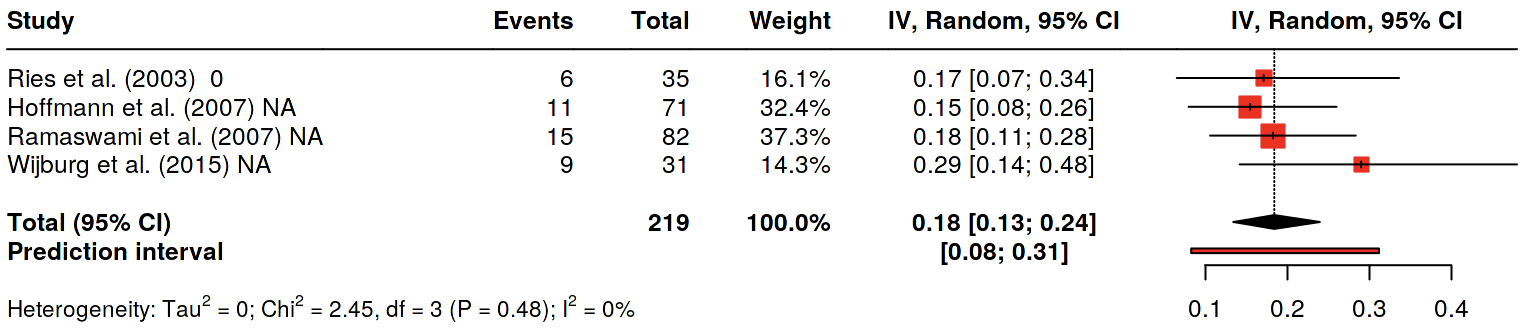

Supplement: Supplementary file 1 [file nutrients-17-01194-s001.zip › supplementary/supplementary figure S4.png]

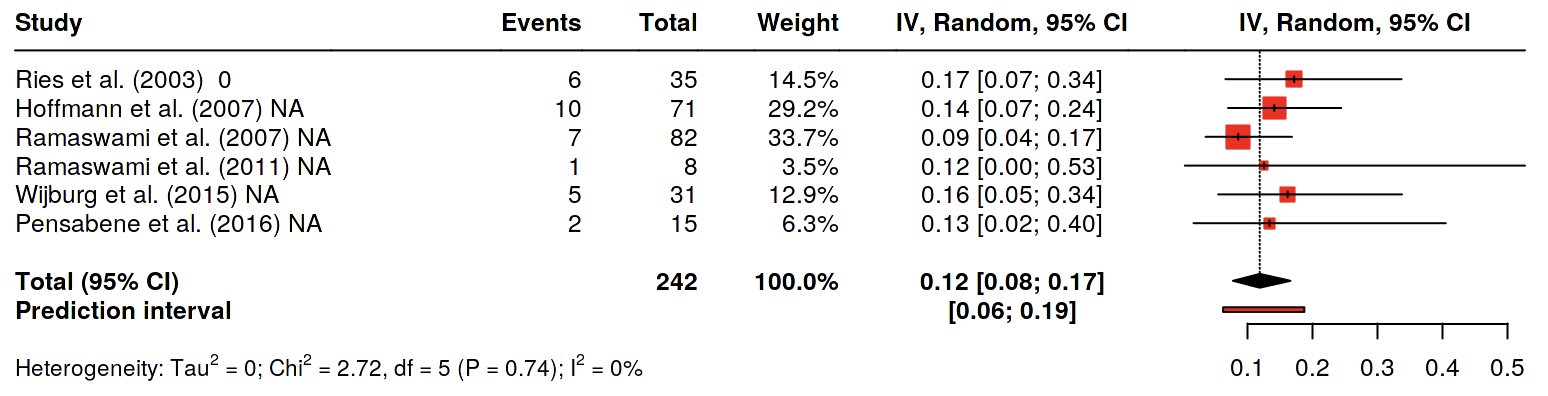

Supplement: Supplementary file 1 [file nutrients-17-01194-s001.zip › supplementary/supplementary figure S5.png]

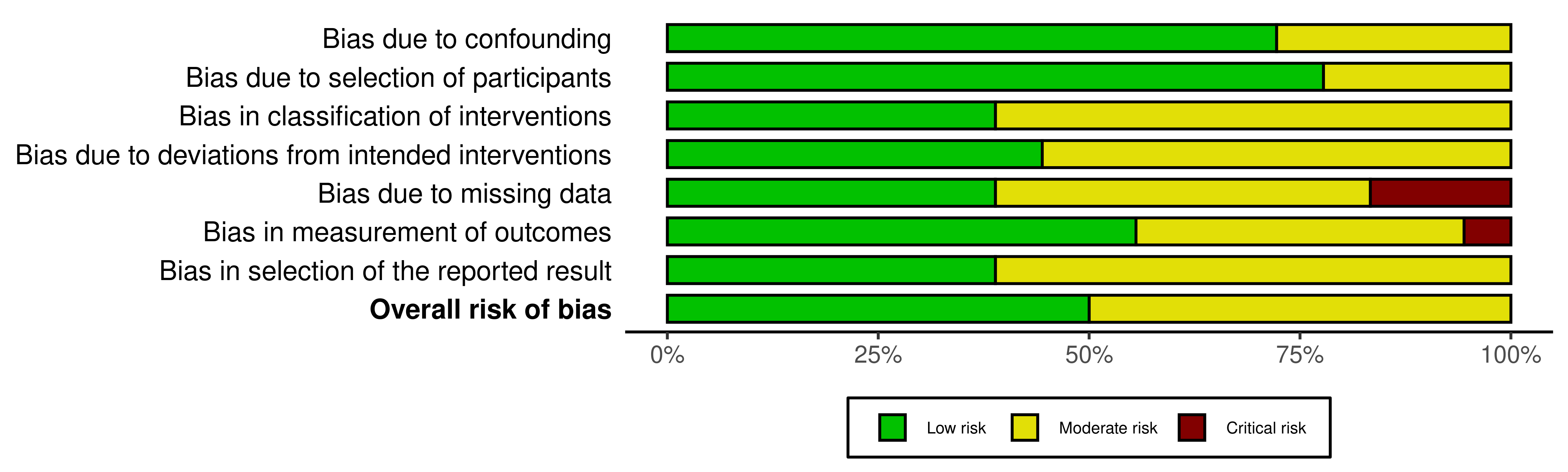

Supplement: Supplementary file 1 [file nutrients-17-01194-s001.zip › supplementary/Supplementary figure S6.png]
